# Dry Needling for Tension-Type Headache: A Scoping Review on Intervention Procedures, Muscle Targets, and Outcomes

**DOI:** 10.3390/jcm14155320

**Published:** 2025-07-28

**Authors:** Ana Bravo-Vazquez, Ernesto Anarte-Lazo, Cleofas Rodriguez-Blanco, Carlos Bernal-Utrera

**Affiliations:** 1 Doctoral Program in Health Sciences, University of Seville, 41009 Seville, Spain; abravovazquez@hotmail.com; 2 Faculty of Health, UNIE University, 28015 Madrid, Spain; 3 Physiotherapy Department, Faculty of Nursing, Physiotherapy and Podiatry, University of Seville, 41009 Seville, Spain; cleofas@us.es (C.R.-B.); cbutrera@us.es (C.B.-U.)

**Keywords:** dry needling, headache, trigger points

## Abstract

**Background/Objectives**: Tension-type headache (TTH) is the most prevalent form of primary headache. The etiology of TTH is not yet fully understood, although it is associated with the presence of myofascial trigger points (MTPs) in cervical and facial muscles. Dry needling (DN) therapy has emerged as an effective and safe non-pharmacological option for pain relief, but there are a lack of systematic reviews focused on its specific characteristics in TTH. The aim of this paper is to examine the characteristics and methodologies of DN in managing TTH. **Methods**: A scoping review was conducted with inclusion criteria considering studies that evaluated DN interventions in adults with TTH, reporting target muscles, diagnostic criteria, and technical features. The search was performed using PubMed, Embase, Scopus, and the Web of Science, resulting in the selection of seven studies after a rigorous filtering and evaluation process. **Results**: The included studies, primarily randomized controlled trials, involved a total of 309 participants. The most frequently treated muscles were the temporalis and trapezius. Identification of MTPs was mainly performed through manual palpation, although diagnostic criteria varied. DN interventions differed in technique. All studies included indicated favorable outcomes with improvements in headache symptoms. No serious adverse effects were reported, suggesting that the technique is safe. However, heterogeneity in protocols and diagnostic criteria limits the comparability of results. **Conclusions**: The evidence supports the use of DN in key muscles such as the temporalis and trapezius for managing TTH, although the diversity in methodologies and diagnostic criteria highlights the need for standardization. The safety profile of the method is favorable, but further research is necessary to define optimal protocols and improve reproducibility. Implementing objective diagnostic criteria and uniform protocols will facilitate advances in clinical practice and future research, ultimately optimizing outcomes for patients with TTH.

## 1. Introduction

Headache is one of the most prevalent disorders worldwide, affecting approximately 2.81 billion people in 2021, with women aged 15 to 49 years being the most impacted [1]. Due to its potential to cause disability and incur high financial costs, headaches have become a significant public health challenge [2].

Headache classification is based on the International Classification of Headache Disorders, 3rd edition (ICHD-3). It categorizes headaches into two main groups: primary and secondary. Primary headaches are disorders in themselves, with no identified cause, while secondary headaches are caused by or occur due to another condition. Primary headache disorders include migraine, tension-type headache (TTH), trigeminal autonomic cephalalgias, and others [3]. Among these, TTH is recognized as the most prevalent form of primary headache, affecting between 26% and 38% of the global population [4]. This disorder manifests as bilateral, dull, non-pulsating, and diffuse pain of mild-to-moderate intensity in the head or neck, which can significantly decrease quality of life and increase disability levels [2,4]. Despite its high incidence, the etiology and underlying mechanisms of TTH remain poorly understood [5]. Although current research suggests that muscle activity and facilitation of nociceptive pain processing play important roles in the pathogenesis of this condition, findings have been controversial [6]. While electromyography studies have reported normal muscle activity in patients with TTH, increased activity has also been observed in myofascial trigger points (MTPs). Therefore, it has been suggested that MTPs may contribute to this condition [5,6,7].

MTPs are defined as irritable points in skeletal muscle that exhibit a palpable hypersensitive nodule within a taut band. They can cause pain, referred tenderness in other areas, and motor dysfunction [8,9]. Recent clinical evidence has demonstrated the presence of MTPs in specific muscles of the neck and shoulders in individuals with TTH, suggesting that these points may contribute to peripheral nociceptive inputs. Specifically, MTPs located in muscles innervated by the C1–C3 nerves and the trigeminal nerve may be responsible for peripheral nociceptive input, generating an afferent overload in the caudal nucleus of the trigeminal nerve. This overload can lead to referred pain in the orofacial region, consistent with the neuronal convergence theory. In this context, hyperexcitability of nociceptive pathways appears to play a crucial role, as sensitization of these pathways may explain the increased muscle sensitivity observed in TTH [8,9,10].

The treatment of TTH includes both pharmacological and non-pharmacological approaches. One non-pharmacological method that has demonstrated a good cost-effectiveness ratio [11], along with low morbidity and mortality [12], is dry needling (DN). DN involves inserting a solid, filiform needle into the skin to target MTPs, aiming to disrupt dysfunctional motor endplates and alleviate neuromusculoskeletal pain [13]. The use of DN has gained traction in clinical practice [14]; however, to date, there has been no review of the literature focusing solely on the characteristics of DN interventions in the context of TTH.

Given these considerations, this review aims to comprehensively analyze the current literature on TTH and its relationship with MTPs, with a particular emphasis on DN interventions. By synthesizing recent research findings, we seek to examine the characteristics and methodologies of DN in managing this prevalent headache disorder.

## 2. Materials and Methods

This scoping review was conducted to explore and synthesize the literature describing the evidence on target muscles and specific intervention characteristics of dry needling in the treatment of tension-type headache. The protocol was developed following the framework proposed by Arksey and O’Malley (2005) and further refined by Levac et al. (2010) [15,16]. To ensure the quality of reporting, the PRISMA extension for Scoping Reviews (PRISMA-ScR) checklist was used [17].

Following Arksey and O’Malley’s five-stage framework [15], the review included the following: (a) identifying the research question; (b) identifying relevant studies; (c) selecting the studies; (d) charting the data; and (e) collating, summarizing, and reporting the results.

### 2.1. Identifying the Research Question

The main research question was as follows: What are the target muscles and specific intervention characteristics of dry needling in patients with tension-type headache, according to the available evidence?

Sub-questions included the following:

- What diagnostic criteria have been used to identify the presence or absence of trigger points in the target muscles for dry needling in patients with tension-type headache?
- What specific methodology has been applied in the administration of dry needling interventions?
- What are the potential adverse effects or unwanted reactions reported following dry needling application in muscles related to tension-type headache?

The study was guided by the Participant–Concept–Context (PCC) framework [18]. The population included adults diagnosed with any form of head and neck pain; the concept was orofacial pain or headache, and the context included any clinical or research setting.

### 2.2. Identifying Relevant Studies

#### 2.2.1. Eligibility Criteria

Inclusion Criteria:

- Studies addressing tension-type headache as the primary diagnosis;
- Studies evaluating dry needling interventions, applied alone or as part of a combined treatment (provided the effect of dry needling is specified);
- Studies reporting the targeted muscles, diagnostic criteria for muscles, and/or characteristics of the dry needling protocol (frequency, duration, technique, adverse effects, or unwanted reactions);
- Articles published in English, French, or Spanish;
- Quantitative or mixed-methods studies: clinical trials, quasi-experimental studies, case reports, and case series;
- No restriction on the year of publication.

Exclusion Criteria:

- Studies that do not clearly differentiate dry needling from other techniques;
- Studies where tension-type headache is not the primary diagnosis or is unspecified;
- Systematic reviews, meta-analyses, letters to the editor, editorials, or commentaries;
- Studies conducted on animals or non-human models.

#### 2.2.2. Information Sources

A comprehensive search was conducted in the following databases: PubMed, Embase, Scopus, and Web of Science. Additionally, references for included studies were manually screened to identify further relevant publications.

#### 2.2.3. Search Strategy

A sensitive and comprehensive search strategy combining controlled vocabulary and free-text terms was developed for each database:

- PubMed: Used MeSH terms (“Dry Needling”[MeSH Terms] OR “Dry Needling”[Title/Abstract]) AND (“Headache, Tension-Type”[MeSH Terms] OR “tension-type headache”[Title/Abstract] OR “headache”[Title/Abstract] OR “cervicogenic headache”[Title/Abstract]) AND (“Myofascial Pain Syndromes”[MeSH Terms] OR “trigger points”[Title/Abstract])
- Embase: Used EMTREE terms and synonyms (‘dry needling’/exp OR ‘dry needling’) AND (‘tension-type headache’/exp OR ‘tension-type headache’ OR ‘headache’ OR ‘cervicogenic headache’) AND (‘myofascial trigger point’/exp OR ‘trigger point’)
- Web of Science: Used the Topic (TS) field for free-text search:
- TS = (“dry needling” OR “intramuscular stimulation”) AND
- TS = (“tension-type headache” OR “tension headache” OR “TTH” OR “headache” OR “cervicogenic headache”) AND
- TS = (“myofascial trigger points” OR “trigger points” OR “myofascial pain”)
- Scopus: TITLE-ABS-KEY(“dry needling” OR “intramuscular stimulation”) AND
- TITLE-ABS-KEY(“tension-type headache” OR “tension headache” OR “TTH” OR “headache” OR “cervicogenic headache”) AND
- TITLE-ABS-KEY(“myofascial trigger points” OR “trigger points” OR “myofascial pain”)

### 2.3. Study Selection

After removing duplicates, titles and abstracts were screened independently by two reviewers (EA and CB). Full-text articles were retrieved for potentially relevant records and assessed against the eligibility criteria. Discrepancies were resolved through discussion or by a third reviewer (CR)

### 2.4. Data Charting

#### 2.4.1. Data Extraction

A standardized form was developed to collect key information from the included studies. Two reviewers independently extracted the data (AB and EA). Any disagreements were resolved by consensus or consultation with a third reviewer (CB).

#### 2.4.2. Extracted Variables

The following data were extracted:

1. Study characteristics: author, year, country, study design;
2. Participant characteristics: headache intensity and location, sample size, age, sex;
3. Target muscles;
4. Diagnostic criteria;
5. Characteristics of dry needling interventions: technique, frequency, duration;
6. Adverse effects or unwanted reactions.

### 2.5. Collating, Summarizing, and Reporting the Results

A descriptive and narrative synthesis of the included studies was conducted, organized by target muscles, diagnostic criteria, and characteristics of the dry needling interventions. No formal risk of bias assessment was performed, consistent with the scoping review methodology.

## 3. Results

**Figure 1:**
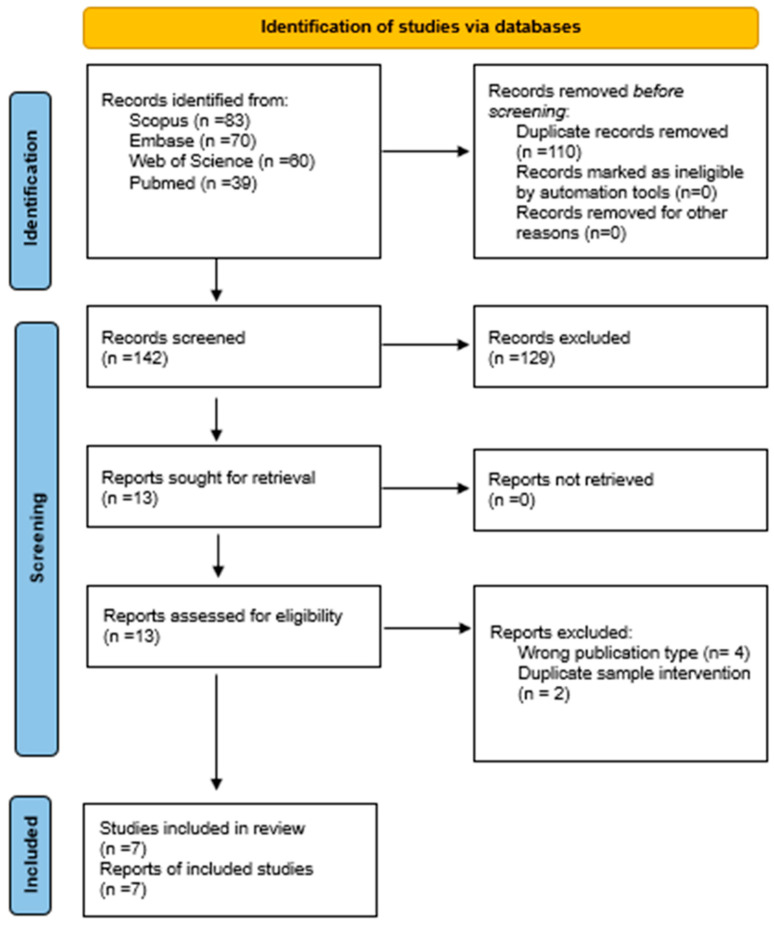
PRISMA flowchart [17].

The search in all databases yielded a total of 252 articles, of which 110 duplicate articles were eliminated and 129 were ineligible. A total of 13 articles were analyzed by title and abstract. Finally, 13 articles were analyzed in full text and 6 articles were excluded because they met the exclusion criteria. See Figure 1.

A total of seven studies were included in this review [19,20,21,22,23,24,25].

### 3.1. Study and Participant Characteristics

Of the 7 included studies, 5 were randomized controlled trials [19,21,23,24,25] and 2 were case reports [20,22]. Overall, the studies reviewed demonstrate a solid methodological level, with generally appropriate randomization procedures and, in some cases, well-implemented blinding strategies. The double-blind trials, such as those conducted by Gildir et al. [21] and Karakurum et al. [24], exhibit the highest methodological quality, while the single-blind studies by Kamali et al. [23] and Monti-Ballano et al. [25] provide an acceptable level of control, albeit with potential risks of bias in outcome assessment. In total, 309 subjects participated.

The characteristics of the participants can be found in Table 1.

#### 3.1.1. Diagnostic Criteria for TTH

One study employed the first edition of the ICHD, three studies utilized the second edition, and two studies applied the third edition. All studies adopted the diagnostic criteria of the International Headache Society that were appropriate to the time of their publication. Only one study did not specify the diagnostic criteria used, reporting instead that the diagnosis was made by a neurologist.

#### 3.1.2. Intensity, Frequency, and Duration of Headache

In the studies analyzed, significant variations were observed in the effects of different interventions on headache intensity, frequency, and duration.

Regarding intensity, most studies reported a significant decrease following treatment. For instance, De Abreu Venancio et al. [19] observed a reduction in the Symptom Severity Index (SSI) across all three treatment groups (G1: 0.5 to 0.3; G2: 0.6 to 0.4; G3: 0.4 to 0.4). Gagnon et al. [20], in a case report, documented a progressive reduction in VAS scores from 6 to 0 over the course of five sessions. Gildir et al. [21] reported a more pronounced decrease in the intervention group (from 4.5 to 0.7) compared to the control group, which showed no improvement (from 4.6 to 4.6). Other studies, such as those by Kamali et al. [23] and Monti-Ballano et al. [25], also reported significant reductions in headache intensity, particularly in groups receiving dry needling therapy.

In terms of headache frequency, most studies recorded substantial decreases. Gildir et al. [21] reported a drop from 18.5 to 3.8 days/month in the treatment group, whereas the control group only declined from 18 to 7.9 days/month. Similarly, Kamali et al. [23] observed a reduction in frequency from 5 to 3.1 days/week in the intervention group. Karakurum et al. [24] reported a high baseline monthly frequency, with a notable reduction following intervention (headache index from 30.4 to 10.8).

With respect to headache duration, only a few studies provided specific data. Gildir et al. [21] documented a reduction in the experimental group from 3.9 to 0.7 h/day after the intervention. Issa and Huijbregts [22], in a case study, reported a variable headache duration ranging from 4 to 72 h. Other studies, including those by Kamali et al. [23], Karakurum et al. [24], and Monti-Ballano et al. [25], did not specify the headache duration.

The characteristics of the studies can be found in Table 1.

### 3.2. Intervention Characteristics

#### 3.2.1. Target Muscles

Most of the studies analyzed included, among others, the temporalis and trapezius muscles as target areas [19,21,22,23,25]. Karakurum et al. also included the splenius capitis and splenius cervicis, in addition to the trapezius muscle [24], while Gagnon et al. focused solely on the levator scapulae muscle [20]. Only the study conducted by Monti-Ballano et al. incorporated all of the muscles known to refer pain to cranial or facial regions [25], which are typically associated with headaches according to Travell and Simons [26]. These muscles include the upper trapezius, splenius capitis and cervicis, semispinalis, rectus capitis posterior major, superior and inferior obliquus capitis, anterior and posterior occipitofrontalis, temporalis, masseter, as well as the clavicular and sternal heads of the sternocleidomastoid, zygomaticus major, and levator scapulae [25].

#### 3.2.2. Diagnostic Criteria

The diagnostic criterion employed in most studies was the presence of active myofascial trigger points (MTPs) that reproduced headache symptoms upon manual palpation. Some studies reported using an algometer for precise digital palpation (1.5 KG pressure) [19,25]. Gildir et al. utilized flat palpation for all muscles except the trapezius, which underwent pincer palpation [21]. Only one study relied solely on the patient’s history to establish the diagnosis [24].

#### 3.2.3. DN Intervention Characteristics

In terms of DN intervention characteristics, some authors noted the use of alcohol to disinfect the skin prior to puncturing [19,21]. Most authors provided details on the dimensions of the needles used, with calibers ranging from 0.2 to 0.3 mm and lengths from 0.13 to 0.5 mm. Some studies discussed patient positioning during the intervention, ranging from supine [22] to prone [20] or sitting [21]. Most authors elaborated on the technique applied, with some explaining the optimal angle for needle insertion [19,20], while others described various DN techniques based on the specific muscle being treated [22,25]. The average number of treatment sessions reported was three, ranging from one to five, with one session noted by Karakurum et al. and five by Gagnon et al. [20,24].

#### 3.2.4. Outcomes

All studies included in this review indicated favorable outcomes from DN interventions, with improvements in headache symptoms being observed across the board. Abreu Venancio et al. further reported that in their study, they achieved more beneficial results with other treatments compared to DN alone, as the injection of substances into the puncture made the process less painful [19].

Gildir et al. [21] explicitly reported that DN was both effective, not only in reducing the frequency, intensity, and duration of headaches, but also in improving health-related quality of life (HRQoL).

#### 3.2.5. Adverse Effects or Unwanted Reactions

None of the studies included in this review reported any notable adverse effects [19,20,21,22,23,24,25].

Table 2 provides a summary of the characteristics of the interventions.

## 4. Discussion

The main aims of this review were to assess the methodologies and clinical characteristics of DN in the management of TTH. To the best of our knowledge, the conducted scoping review has been the first to provide an expanded view of the characteristics of DN interventions in the treatment of TTH, highlighting both the target muscles and the diagnostic criteria, the specifics of the intervention, and the associated adverse effects. The comprehensive approach offered by this review and the relevant aspects it emphasizes contribute to the understanding of this technique and its clinical application.

The analysis of the included studies shows a clear predominance in the selection of the temporalis and trapezius muscles, with up to five studies implementing these interventions [19,21,22,23,25], corroborating previous findings regarding their relevance in the occurrence of MTPs related to this disorder [27,28]. This parallelism with the existing literature validates the focus on target muscles adopted in most of the analyzed studies and establishes a solid foundation for future research. However, a wide diversity is observed in the approach to other involved muscles. In other words, numerous muscles can independently or collectively trigger pain, and the different existing theories and methodologies lead to focusing on one muscle group or another. This can be understood, for example, through the study by Karakum et al. [24], which, in addition to the trapezius, concentrates on the splenius capitis and cervicis, following the radiculopathy origin described by Gunn, who attributes the pain to the paravertebral musculature [29]. Conversely, Gagnon et al. focus exclusively on the levator scapulae as the trigger point for headache in a unique case of TTH with levator scapulae syndrome, emphasizing a more isolated origin related to MTPs [20]. It is also important to highlight the study by Monti-Ballano et al., which was the only one to encompass all muscles known to refer pain to the facial or cranial regions, offering a crucial opportunity to unify criteria [25]. These results suggest, in line with the findings of Fernández-de-las-Peñas et al., the need for standardization in the selection of target muscles for DN in future research. Such standardization would not only facilitate the reproducibility of results across different contexts but also potentially enhance our understanding of the etiology and mechanisms underlying TTH [27,28]. This underlines the necessity of rigorous clinical reasoning when selecting muscles, techniques, treatment dosage, and patient profiling (e.g., chronic vs. episodic TTH, central sensitization status) [30].

Regarding the diagnostic criteria employed, there was a trend in six of the seven included studies towards the identification of active MTPs that reproduce TTH symptoms through manual palpation [19,20,21,22,23,25]. The variability in the palpation techniques used, ranging from digital palpation with the use of algometers [19,25] to flat palpation [21], reflects the heterogeneity in diagnostic approaches. This ambiguity may significantly influence the interpretation of the observed results, as they largely depend on the palpation process itself and the clinician’s experience. This may lead to discrepancies between the approach of different physiotherapists or even difficulties in making a differential diagnosis of other muscular injuries. Furthermore, although MTPs are defined as hyperirritable areas within a muscle that can cause local and referred pain, there is no universal consensus on the specific criteria for defining an MTP, which further complicates their diagnosis. Pain and tenderness upon palpation, as well as the interpretation of clinical signs such as the presence of palpable nodules, are highly subjective criteria that can vary greatly, potentially affecting diagnostic accuracy and, consequently, treatment effectiveness. Therefore, it is essential to address the need for establishing more standardized protocols with more objective criteria to improve the reliability of trigger point identification [31,32].

In terms of the characteristics of DN intervention, there was a consensus on the use of disinfectants and the specification of needle dimensions, reinforcing an essential aspect: the importance of safety and technical quality during the procedure. The studies employed different types of DN techniques, but all were based on the methodology described by Travell and Simons [26]. On one hand, Issa and Huijbregt described a meticulous process following the guidelines of the American Physical Therapy Association [33]. They emphasized securing the tube in the suspected area, directing the needle according to the muscle, and patient positioning. Additionally, they highlighted precautions to avoid injury to nearby structures such as the lung, carotid artery, facial nerve, and temporal artery [22]. Monti-Ballano et al., for their part, also adopted a technique based on muscle size and the presence of dangerous structures, using inward puncture and bidirectional rotation in muscles with flat bellies or proximity to vessels and nerves [25]. This underscores the need for precise knowledge of human anatomy and careful attention to safety during the intervention to prevent complications, as reported by Lee et al. in their study [34]. Abreu Venancio et al., in their study, used a multi-directional technique (up, down, lateral, and medial), repeating the puncture until the desired response was achieved, as indicated by Hong’s technique [35]. They emphasized the importance of mechanical disruption of the MTPs for treatment success, although they did not specify patient positioning or the exact technique [19]. Gagnon et al. described a more detailed technique, with the patient in the prone position, using the clamp grip and fan technique to target different points within the muscle, as well as performing periosteal tapping at the enthesis [20], a technique previously used in various musculoskeletal pathologies but with the same therapeutic approach, as seen in the studies reviewed by Tought et al. [36]. The technique of rolling or twisting the needle was also mentioned by Gagnon et al. to enhance physiological effects [20], based on the method described by Gunn [29]. On the other hand, some studies, such as those by Gildir et al. and Karakum et al., relied on specific techniques with needle retention times of 20 to 30 min [21,24]. Kamali et al. indicated that their employed technique was based on the methodology described by Demerholl and Fernandez de las Peñas in 2013 [37], although they provided few details about patient positioning or the exact technique [23]. This diversity of techniques across the different studies raises the question of whether it could affect treatment efficacy or be relevant to the outcomes. However, there are studies comparing these techniques, such as that by Taşoğlu et al., that compared a more static approach, where the needle remains inserted in the treatment area for a few minutes, versus a more dynamic approach, where the needle is repeatedly inserted in multiple directions. They concluded that both techniques were equally effective [38]. In summary, the literature demonstrates the use of a flexible and context-dependent strategy tailored to different muscles, suggesting potential variability in terms of application.

The studies reviewed show that the intervention with DN in MTPs has a positive impact, not only on the intensity and frequency of headache, but also on HRQoL and, by extension, on people’s ability to perform tasks of daily living. Gildir et al. [21] explicitly reported that DN was effective in improving HRQoL and this improvement suggests a favorable functional impact, enabling patients to resume their daily activities with greater ease. Although the remaining studies did not report direct measures of quality of life, the reduction in pain indicates a considerable positive effect on overall well-being and daily functioning, suggesting a likely recovery of the patients’ functional capacity in everyday tasks.

An encouraging aspect highlighted in this review is the scarcity of significant adverse effects associated with DN, which is a crucial point that should be communicated to gain acceptance for this technique as a safe treatment option for patients with TTH. However, the absence of reported adverse effects does not necessarily indicate that there is no risk; therefore, it is important for future research to collect data on adverse effects to provide a clearer picture of the safety of this treatment.

Another important finding of this review is the potential of DN as a therapeutic intervention that can be tailored to the specific clinical characteristics of patients with TTH, both in its episodic and chronic forms. Most of the included studies focus on heterogeneous populations, without a clear distinction between these subtypes, which limits the ability to generate specific, evidence-based recommendations for clinical practice. However, given the differing pathophysiology between episodic and chronic TTH, it would be reasonable to consider personalizing DN interventions based on headache subtypes. For instance, patients with chronic TTH may benefit from more intensive or extended protocols, involving a greater number of sessions or combining DN with other therapeutic modalities (such as therapeutic exercise or pain management education). In contrast, in episodic cases, brief and targeted interventions may be sufficient to reduce recurrence or prevent chronification.

The scoping review presented here has several limitations. Firstly, the small number of included studies restricts the breadth of the analysis and may limit the generalizability of the findings. Additionally, there was notable heterogeneity among the studies in terms of methodologies and intervention protocols, which complicates direct comparisons and synthesis of results. Furthermore, many studies lacked comprehensive reporting of key details such as specific technical procedures, which hampers the ability to draw definitive conclusions and identify best practices. With respect to diagnostic classification, the use of different editions of the International Classification of Headache Disorders (ICHD) across the included studies may significantly contribute to the clinical heterogeneity observed among the samples. The evolution of diagnostic criteria—from the broader descriptions in ICHD-1 to the more refined and flexible definitions in ICHD-3—implies that participants diagnosed with tension-type headache under different editions do not necessarily represent homogeneous populations. This variability can influence the inclusion of clinically distinct subtypes (episodic vs. chronic), the presence or absence of symptoms such as photophobia or phonophobia, and the diagnostic threshold itself. Moreover, the lack of specification regarding the diagnostic criteria used in some studies introduces an additional risk of selection bias and limits the reproducibility of the findings. Therefore, these factors must be carefully considered when interpreting the consistency and applicability of the results. Lastly, the diagnostic process for identifying MTPs is largely subjective and lacks standardization across studies, which may compromise the reliability of findings and limit their clinical applicability.

Given these limitations, it is recommended that future studies be conducted to incorporate new and more robust evidence as it becomes available, thereby strengthening the current findings. Moreover, future research should focus on the subclassification of patients based on the specific muscles affected, as this could allow for more tailored and effective intervention strategies. Standardizing intervention protocols across studies would also facilitate more meaningful comparisons and meta-analyses. Additionally, longitudinal studies with complete and detailed reporting are needed to better understand the techniques. These lines of investigation could significantly advance the field by providing clearer guidelines and improving clinical outcomes.

## 5. Conclusions

This scoping review has provided a comprehensive overview of the methodologies and clinical characteristics of dry needling (DN) in the management of tension-type headache (TTH), highlighting key aspects such as the target muscles, diagnostic criteria, techniques used, and potential adverse effects. A predominant trend was observed in the selection of the temporalis and trapezius muscles, supported by the previous literature, which reinforces the importance of these muscles in the pathophysiology of TTH. However, significant heterogeneity was also noted in the choice of other muscles, as well as in the techniques and protocols employed, reflecting the diversity of approaches and theories currently in practice. Variability in diagnostic criteria, especially in identifying active trigger points through palpation, along with the lack of standardized protocols, complicates result comparison and the reproducibility of interventions. Additionally, although most studies reported few adverse effects, the limited documentation regarding potential risks underscores the need for increased attention to be paid to safety and systematic data collection in future research.

## Figures and Tables

**Table 1:** Study and participant characteristics.

Author, Year, Country	Study Design	Sample Size (N) Gender (F/M) Age	Intensity, Frequency and Duration Headache	Location Headache
De Abreu Venancio et al., 2009 Brazil [19]	RCT (G1: dry needling G2: lidocaine G3: botulinum toxin)	N = 45 40 F/5 M 18–65 years	Symptom Severity Index (SSI) = three subscales of pain: frequency, intensity, duration. SSI (pretreatment–posttreatment): G1: 0.5-0.3 G2: 0.6-0.4 G3: 0.4-0.4	Orofacial or cervical region
Gagnon et al., 2024 USA [20]	A case report	N = 1 1 M/0 F 63 years	Intensity (cm of VAS): Pretreatment: 6 Posttreatment session 1: 3 Posttreatment session 3: 1 Posttreatment session 5: 0 Frequency: Not specified Duration: Not specified	Near the medial aspect of the superior angle of the scapula, bilaterally and progressively radiating upward toward the occiput
Gildir et al., 2019 Turkey [21]	RCT (IG: dry needling CG: sham dry needling)	IG: N = 80 41 F/39 M 36.7 years CG: N = 80 44 F/39 M 36 years	(Pretreatment- posttreatment- follow up) Intensity (cm of VAS): IG: 4.5-0.7-0.9 CG: 4.6-4.6-4.9 Frequency (days/month): IG: 18.5-3.8-4.9 CG: 18-7.9-16.3 Duration (hours/day): IG: 3.9-0.7-0.7 CG: 3.8-3.9-4.1	Not specified
Issa et Huijbregts, 2006 USA [22]	A case report	N = 1 1 F/0 M 48 years	Intensity (pretreatment–posttreatment): severe–mild Frequency (pretreatment–posttreatment): 1/week- 1 to 4/month Duration: 4–72 h	Bilateral frontal head region, left cheek and jaw region, bilateral suboccipital, lower neck, and left back of neck
Kamali et al., 2019 Iran [23]	RCT (IG: dry needling CG: friction massages)	IG: N = 20 16 F/4 M 37.4 years CG: N = 20 19 F/1 M 33.7 years	Intensity (pretreatment–posttreatment): IG: 8-5 (VAS) CG: 9.5-5.3 (VAS) Frequency (pretreatment–posttreatment): IG: 5-3.1 (day/week) CG: 7-4.2 (day/week) Duration: not specified	Not specified
Karakurum et al., 2001 Turkey [24]	RCT (IG: dry needling; CG: subcutaneous insertions)	IG: N = 15 15 F/0 M 28.4 years CG: N = 15 15 F/0 M 27.9 years	Frequency: IG: 29.6 days/month CG: 25.2 days/ month Headache index = intensity × days (pretreatment–posttreatment): IG: 30.4-10.8 CG: 37.4-15.4 Muscle tenderness = palpating the neck muscles (pretreatment–posttreatment: IG: 1.67-0.6 CG: 1.67-1.47 Duration: not specified	Not specified
Monti-Ballano et al., 2019 Spain [25]	RCT	IG: N = 16 31.7 years CG: N = 16 41.4 years	Frequency: IG: 13.7 days/month CG: 13.2 days/month Intensity (pretreatment–posttreatment): IG: 19.2-10.5 (VAS) CG: 19.4-27.9 (VAS) Duration: not specified	Not specified

CG: control group; F: female; IG: intervention group; M: male; RCT: randomized controlled trial; VAS: Visual Analog Scale.

**Table 2:** Intervention characteristics.

Study	Target Muscles	Diagnostic Criteria	Dry Needling Interventions Characteristics	Outcomes	Adverse Effects or Unwanted Reactions
De Abreu Venancio et al., 2009 [19]	Masseter, temporalis, occiput, and trapezius	Anamnesis and a physical exam to confirm the diagnosis of myofascial pain and headache. MTPs were located using digital palpation and the clinical exam was calibrated using a pressure algometer (1.5 kg of pressure)	Skin was cleansed with alcohol; the clinician inserts the needle 1–2 cm away from the trigger point, so that the needle may advance into the trigger point at an acute angle of 30 degrees to the skin	All the groups showed favorable results. The use of lidocaine or botulinum toxin made the technique less painful	Did not register any serious adverse events
Gagnon et al., 2024 [20]	Levator scapulae	Pincer grasp palpation to the distal levator scapulae muscles bilaterally	The patient was in a prone position with his arm internally rotated behind the back and a rolled towel was placed under the anterior shoulder. The needles were inserted obliquely from lateral to medial, superior to inferior, and posterior to anterior through the upper trapezius and into the levator scapulae muscle belly. Needle dimensions: 0.30 × 50 mm 5 sessions/2 months	Improvement in the patient’s headache symptoms	Not specified
Gildir et al., 2019 [21]	Masseter, temporalis, frontalis, splenius, upper trapezius, and suboccipital	Patients with active MTPs (referred pain after 10 s of palpation of the muscle). Pincer palpation method for upper trapezius muscle Flat palpation method for other muscles	Firstly, the area was cleaned with alcohol; then, with patients sitting, the needle was inserted into active MTPs for 20 min. Needle dimensions: 0.25 × 40 mm/0.25 × 25 mm 3 sessions per week for 3 weeks	Dry needling was effective and safe in reducing headache frequency, intensity, and duration, and increasing health-related quality of life	Five of the patients in each group experienced pain and fear during the procedure
Issa et Huijbregt, 2006 [22]	Upper trapezius, sternocleidomastoid, splenius capitis, suboccipital, masseter, and temporalis	Active MTP were diagnosed by way of subjective history, neck mobility tests, and manual palpation	The needle was fixed in the suspected area using a pincer grip or flat palpation depending on the muscle orientation, location, and direction of penetration; the needle was gently loosened from the tube and then a flick or tap of the top of the needle was performed to quickly penetrate the layers of the skin; the needle was then guided towards the taut band until resistance was felt in a particular direction and deep, gentle, small-amplitude withdrawals and penetrations of the needle were performed until a trigger point zone was reached; the needle was removed once palpable and/or visible release of the taut band had been determined. Needle dimensions: 0.3 × 30 mm/0.3 × 50 mm/0.2 × 13 mm 3 sessions/week for 6 weeks	Participant was reported to do very well with no headache	Not specified
Kamali et al., 2019 [23]	Sub-occipital, temporalis, sternocleidomastoid, and upper trapezius	Active MTPs were diagnosed by the presence of a taut band and the jump sign	Specific position for needling each muscle, and needling was carried out directly on MTPs. 3 sessions for 1 week	The frequency and intensity of headaches improved significantly in both study groups. IG increased the pain threshold significantly more than CG	Not specified
Karakurum et al., 2001 [24]	Splenius capitis, splenius cervicis, and mid-trapezius	Diagnosis was based upon the history given	The needles were left inserted in the muscle for 30 min. Needle dimensions: 0.3 × 25.4 mm 1 session/week, 4 weeks	There was significant improvement in IG compared with CG in muscle tenderness	Not specified
Monti-Ballano et al., 2019 [25]	Upper trapezius, splenius capitis and cervicis, semispinalis, rectus capitis posterior major, superior and inferior obliquus capitis, posterior and anterior occipitofrontalis, temporalis, masseter, clavicular and sternal head of sternocleidomastoid, zygomaticus major, and levator scapulae	MTP was considered active if the pain elicited during the pressure replication (using digital algometer) reproduced at least a portion of the TTH pain pattern typically reported by the patient	Specific patient position and needle insertion adapted to the muscle. In-and-out needling or needle winding in muscles with a large-diameter muscular belly and no nearby dangerous structures, and the bidirectional rotation technique was employed in muscles with a very flat muscular belly and those with structures like blood vessels and nerves nearby. Needle dimensions: 0.32 × 40 mm/0.32 × 25 mm 3 sessions	In IG, the intensity (VAS) significantly decreased	Did not register any serious adverse events

CG: control group; IG: intervention group; MTPs: myofascial trigger points; VAS: Visual Analog Scale.

## Data Availability

The authors declare no new data.
